# Interleukin-17A Contributes to Bacterial Clearance in a Mouse Model of Streptococcal Toxic Shock-Like Syndrome

**DOI:** 10.3390/pathogens10060766

**Published:** 2021-06-17

**Authors:** Lei Xu, Xi Lu, Peng Xiao, Ran Liu, Kun-Long Xia, Mei-Zhou Wu, Mei-Lin Jin, An-Ding Zhang

**Affiliations:** 1 State Key Laboratory of Agricultural Microbiology, College of Veterinary Medicine, Huazhong Agricultural University, Wuhan 430070, China; leixu@webmail.hzau.edu.cn (L.X.); lucylook5@webmail.hzau.edu.cn (X.L.); xp199508@webmail.hzau.edu.cn (P.X.); liuran-sky@webmail.hzau.edu.cn (R.L.); xkl@webmail.hzau.edu.cn (K.-L.X.); wumeizhou@mail.hzau.edu.cn (M.-Z.W.); jml8328@126.com (M.-L.J.); 2 Key Laboratory of Preventive Veterinary Medicine in Hubei Province, The Cooperative Innovation Center for Sustainable Pig Production, Wuhan 430070, China; 3 Key Laboratory of Development of Veterinary Diagnostic Products, Ministry of Agriculture of the People’s Republic of China, Wuhan 430070, China; 4 International Research Center for Animal Disease, Ministry of Science and Technology of the People’s Republic of China, Wuhan 430070, China

**Keywords:** *Streptococcus suis*, Interleukin-17A, streptococcal toxic shock-like syndrome, bacterial clearance, neutrophils, antimicrobial proteins

## Abstract

*Streptococcus suis (S. suis)*, an emerging zoonotic pathogen, can cause streptococcal toxic shock-like syndrome (STSLS) in humans with high mortality. STSLS is characterized by high bacterial burden, an inflammatory cytokine storm, multi-organ dysfunction, and ultimately acute host death. Although it has been found that a significantly high level of IL-17A was induced in an NLRP3-dependent manner during STSLS development, the role of IL-17A on *S. suis* STSLS remains to be elucidated. In this study, we found that the epidemic strain SC 19 caused a significantly higher level of IL-17A than the non-epidemic strain P1/7. In addition, higher bacterial burden was observed from SC 19-infected il17a−/− mice than il17a+/+ mice, although acute death, tissue injury and inflammatory cytokines storm were observed in both types of mice. Furthermore, compared with il17a+/+ mice, the level of neutrophils recruitment was lower in il17a−/− mice, and the levels of induced antimicrobial proteins, such as CRAMP, S100A8 and lipocalin-2, were also decreased in il17a−/− mice. In conclusion, this study demonstrated that IL-17A does not contribute to the severe inflammation, although it may play a minor role for bacterial clearance by inducing antimicrobial proteins and promoting neutrophil recruitment during STSLS.

## 1. Introduction

*Streptococcus suis* (*S. suis*) is an important swine pathogen that causes severe economic losses to the porcine industry and is also considered as an emerging zoonotic pathogen which represents a significant threat to human health [1,2,3]. So far, more than 1600 human *S. suis* infections have been reported worldwide [4], and *S. suis* infection has been identified as the leading and second cause of adult meningitis in Vietnam and Thailand [2]. *S. suis* infection mainly induces meningitis, septicemia, arthritis, endocarditis, pneumonia and endophthalmitis, and the pooled case-fatality rate is 12.8% [4]. However, the two large-scale outbreaks of human *S. suis* epidemics in China (the first time had 25 cases with 14 deaths in Jiangsu in 1998, and the second time had 204 cases with 38 deaths in Sichuan in 2005), which led to the unusual development of streptococcal toxic shock-like syndrome (STSLS), with high mortality, raised serious concerns for global public health and challenged the conventional conception of *S. suis* infections being sporadic in humans [2,5,6]. 

STSLS is characterized by high bacterial burden, an inflammatory cytokine storm, multi-organ dysfunction, and ultimately acute host death [5]. Our previous study indicated that the epidemic *S. suis* SC 19 that induced a high expression of SLY, with high membrane perforation activity, which caused several events, including cytosolic K+ efflux, and NLRP3 inflammasome hyperactivation, was essential for the induction of cytokines storm and STSLS in a mouse model [7]. The conclusion was also confirmed by another group [8]. However, how NLRP3 hyperactivation caused STSLS remains to be elucidated.

A significantly high level of IL-17A was induced in an NLRP3-dependent manner during STSLS development [7,9,10], which prompted us to consider the role of IL-17A on STSLS [11]. The potential function of IL-17 on acute infection was to: (1) promote neutrophil recruitment for microorganism clearance [12,13], (2) stimulate the production of antimicrobial proteins (e.g., S100A8, lipocalin-2, and CRAMP) for bacterial killing [11,14,15,16,17], (3) promote the expression of proteins maintaining intestinal epithelial integrity for limiting infection spreading [18,19], (4) induce matrix metalloproteinases for tissue damage [18], and (5) downregulate and reorganize tight junction molecules for BBB disruption and then cause CNS diseases [11,20,21,22]. This means that IL-17A may potentially play roles on *S. suis* clearance or in strengthening inflammation, so whether it is beneficial or inhibitory for the development of STSLS is unclear. Therefore, the present study was to evaluate the role of Interleukin-17A signaling during STSLS development in a mouse model.

## 2. Results and Discussions

### 2.1. IL-17A Was Induced during STSLS

**Figure 1:**
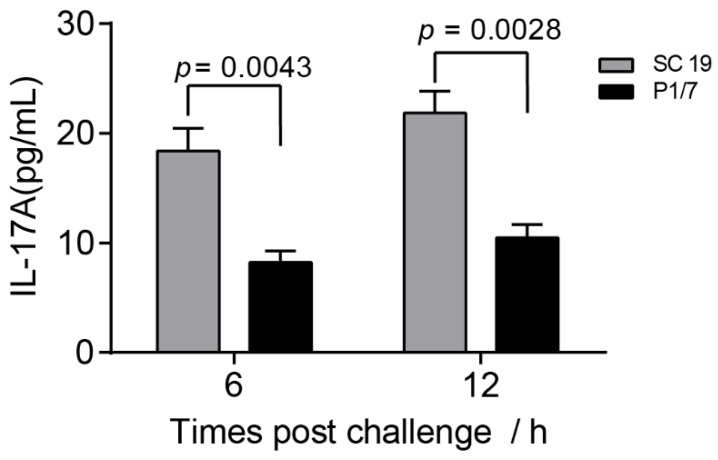
The level of IL-17A caused by the epidemic strain SC 19 was significantly higher than that of the non-epidemic strain P1/7. Wild-type (WT) mice were intraperitoneally infected with *S. suis* SC 19 or P1/7. The level of IL-17A in the blood were determined at the indicated time points (two-tailed, unpaired *t*-tests, *n* = 5).

The previous study indicated that IL-17A was induced in an NLRP3-dependent manner during STSLS [7]. To evaluate the potential role of IL-17A on STSLS, we first compared the IL-17A level induced by the epidemic strain SC 19, which can cause STSLS, and a non-epidemic strain P1/7, which cannot cause STSLS but can cause meningitis. The epidemic strain SC 19 caused a significantly higher level of IL-17A than the non-epidemic strain P1/7 (Figure 1), suggesting that IL-17A was significantly induced during STSLS.

### 2.2. Knockout of il17a Could Not Decrease Mortality during STSLS

STSLS, caused by the epidemic *S. suis* strain, is characterized by high bacterial burden, an inflammatory cytokine storm, multi-organ dysfunction, and ultimately acute death [5]. Our previous study demonstrated that NLRP3 inflammasome was indispensable for the development of STSLS. However, how inflammasome activation contributes to STSLS still remains to be elucidated. Since IL-17A was induced in an NLRP3-dependent manner during STSLS, and IL-17A might have a potential role for bacterial invasion, we considered the role of IL-17A induction by NLRP3 on the severe inflammation.

**Figure 2:**
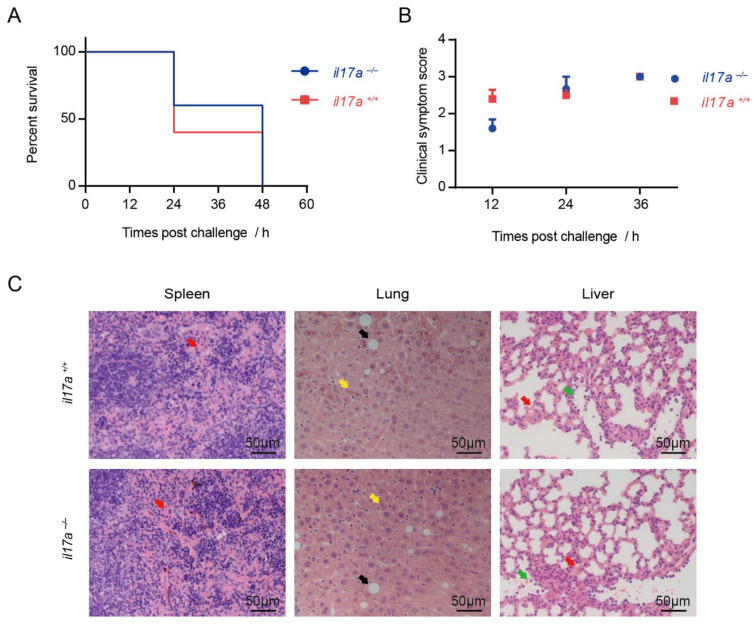
Knockout of il17a could not decrease mortality and alleviate the tissues injury during STSLS. The il17a-deficient mice (il17a−/−) and the wild-type mice (il17a+/+) were intraperitoneally infected with *S. suis* SC 19. (**A**) Survival of mice infected with *S. suis* (log-rank test, *n* = 10). (**B**) Clinical symptom scores of mice infected with *S. suis* (two-way RM ANOVA, *n* = 10). (**C**) H&E staining of infected tissue sections from mice at 12 h post-infection with *S. suis*. Congestion in spleen and lung is indicated by a “red arrow”; necrosis in the liver is indicated by a “yellow arrow”; vacuolated degeneration in the liver is indicated by a “black arrow”; infiltration of inflammatory cells in the lung is indicated by a “green arrow”. Scale bar indicates 50 μM. Error bars represent the mean ± standard deviations.

To directly evaluate the role of IL-17A on STSLS, il17a+/+ and il17a−/− mice were infected with the epidemic *S. suis* strain SC 19. As described before, the infection caused all il17a+/+ mice to die within two days (Figure 2A); however, the infection also caused all il17a−/− mice to die within two days (Figure 2A). In addition, the infection caused il17a+/+ and il17a−/− mice to exhibit similar clinical symptoms (Figure 2B). These results indicated that IL-17A, as a downstream of NLRP3 inflammasome, might not contribute to the acute death caused by the epidemic *S. suis* strain.

### 2.3. Knockout of il17a Could Not Alleviate the Tissues Injury during STSLS

Infection of mice with the epidemic strain could cause acute tissue injury [7], such as severe congestion in the spleen, severe necrosis and vacuolated degeneration in the liver, and severe congestion and dense infiltration of inflammatory cells in the lung, which were also observed in the infection on il17a+/+ mice in the present study (Figure 2C). It is not surprising that infection in il17a−/− mice could not alleviate the tissue injury during STSLS (Figure 2C). As dysfunctions of multiple organs are the major reason for acute death during STSLS, the similar organ damage in il17a+/+ and il17a−/− mice indicated that IL-17A may not play an essential role in the dysfunction of multiple organs, which is an important reason for acute death during STSLS.

### 2.4. Knockout of il17a Strengthened Serum Inflammatory Cytokines Storm

**Figure 3:**
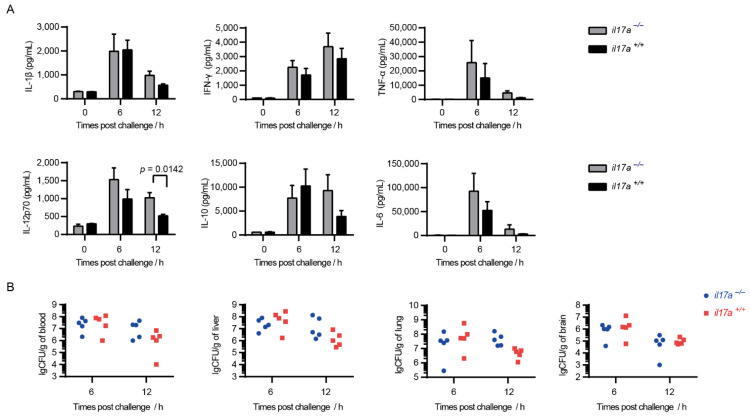
IL-17A could not decrease the cytokine storm during STSLS but was beneficial for *S. suis* clearance at 12 h post-infection. The il17a-deficient mice (il17a−/−) and the wild-type mice (il17a+/+) were intraperitoneally infected with *S. suis* SC 19. (**A**) Cytokine levels in the blood at the indicated time points were determined (two-tailed, unpaired *t*-tests, *n* = 5). (**B**) The bacterial burden in the blood, liver, lung, and brain at the indicated time points were determined (two-tailed, unpaired *t*-tests, *n* = 5). Scale bar indicates 50 μM. Error bars represented the mean ± standard deviations.

Infection of the epidemic strain can lead to inflammatory cytokines storm in mice, and can then further induce dysfunction of multiple organs, eventually leading to acute death. The infection model was confirmed in the present study (Figure 3A). As expected, the knockout of il17a failed to decrease the serum inflammatory cytokines storm, and even induced higher serum inflammatory cytokines, such as IL-1β, IFN-γ, IL-12p70, and IL-10 at 12 h post-infection (Figure 3A). It indicated that IL-17A could not decrease the cytokine storm during STSLS.

### 2.5. IL-17A Is Beneficial for S. suis Clearance at 12 h of Post-Infection

Infection of the epidemic strain could cause mice to exhibit high bacterial burden in various tissues, which was also observed in the present study (Figure 3B). Although IL-17A was confirmed to be beneficial for the invasion of various pathogens, the knockout of il17a could not decrease the bacterial burden in various tissues (Figure 3B). However, the knockout of il17a seemed to cause more bacterial load in the blood at 12 h post-infection (Figure 3B), which might explain the high inflammatory cytokine response at 12 h post-infection (Figure 3B). More interesting, IL-17A was also induced to a high level at 12 h post-infection (Figure 1). It indicated that IL-17A could take an effect for *S. suis* clearance during STSLS.

### 2.6. IL-17A Signaling Contributes to Activation of Neutrophils for Controlling Infections

The infection of mice with the epidemic strain SC 19 could induce a high level of IL-17A at 12 h post-infection (Figure 1). Interestingly, the bacterial burden in blood at that time point was decreased in il17a+/+ but not in il17a−/− mice (Figure 3B), suggesting that IL-17A might contribute to the resistance to *S. suis* infection.

**Figure 4:**
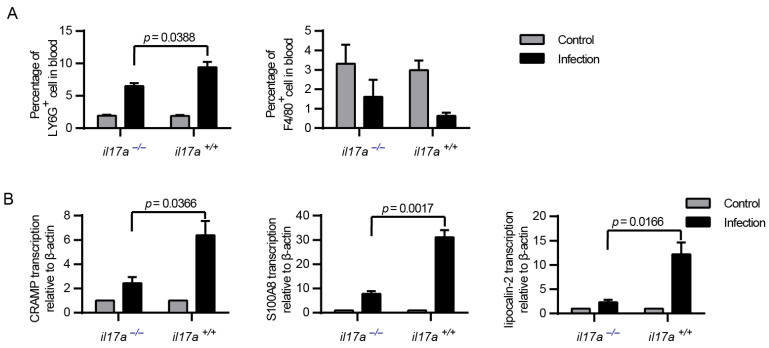
IL-17A could contribute to the production of antimicrobial peptides and the recruitment of neutrophils. The il17a-deficient mice (il17a−/−) and the wild-type mice (il17a+/+) were intraperitoneally infected with *S. suis* SC 19. At the indicated time points, all mice were euthanized by carbon dioxide inhalation to collect the blood via cardiac puncture. (**A**) Flow cytometric analysis of leukocytes in blood (two-tailed, unpaired *t*-tests, *n* = 5). (**B**) Real-time PCR analysis of CRAMP, S100A8 and lipocalin-2 transcription in blood. β-actin was used as the internal control (two-tailed, unpaired *t*-tests, *n* = 3). Neutrophils: phycoerythrin (PE)-conjugated anti-mouse Ly6G, monocytes: fluorescein isothiocyanate (FITC)-conjugated anti-mouse F4/80. Error bars represent the mean ± standard deviations.

The proceeding IL-17A function indicated that IL-17A might promote neutrophil recruitment for microorganism clearance [12,13], or stimulate the production of antimicrobial proteins for bacterial killing [11,14]. As neutrophils were essential for *S. suis* clearance in vivo, the decreased level of neutrophils recruitment could provide an explanation for the decreased resistance of il17a−/− mice to *S. suis* (Figure 4A). In addition, the levels of induced antimicrobial proteins, such as CRAMP, S100A8 and lipocalin-2, also decreased in il17a−/− mice (Figure 4B), suggesting that the induction of IL-17A could be beneficial to bacterial clearance by inducing antimicrobial proteins and promoting neutrophil recruitment, at least at the detected time point.

## 3. Materials and Methods

### 3.1. Bacterial Strain and Culture Conditions

The epidemic strain of *S. suis* serotype 2, SC-19, belongs to ST7, which shows high pathogenicity in humans, mice and pigs [23]. The non-epidemic strain of *S. suis* serotype 2, P1/7, belongs to ST1, which induces only sporadic cases of meningitis and septicemia in pigs [24]. *S. suis* was grown in tryptic soy agar (TSA) (Difco, Detroit, MI, USA) or tryptic soy broth (TSB) (Difco, Detroit, MI, USA) plus 10% newborn bovine serum at 37 °C.

### 3.2. Experimental Infections of Mice with Streptococcus suis

Five- to six-week-old C57BL/6 (il17a+/+) mice and/or il17a−/− mice (purchased from Huazhong Agricultural University) with similar body weights were randomly grouped and challenged with 0.5 mL of *S. suis* strains SC 19 (8 × 108 CFU/mL) or phosphate-buffered saline (PBS) by intraperitoneal (i.p.) injection to evaluate the pathogenicity of *S. suis*.

All mice were monitored three times a day and for a total of seven days for mortality and clinical signs. The clinical scores were assigned as follows: 0 = no symptoms and rapid response to external stimuli; 1 = ruffled coat and slow response to external stimuli; 2 = respond only to repeated stimuli; 3 = no response to external stimuli, or neurological symptoms, etc.; and 4 = dead. Mice exhibiting extreme lethargy or neurological symptoms were considered moribund and were humanely euthanized.

### 3.3. Measurement of Cytokine Response and Bacterial Burden

In addition to the evaluation of mortality, experimental infections were also performed to evaluate the cytokine response and bacterial burden during *S. suis* infection [25]. At the indicated time points, all mice were euthanized by carbon dioxide inhalation, and blood was collected via cardiac puncture. Fifty microliters of blood were serially diluted and then plated on TSA plates to evaluate the bacterial load. Two hundred microliters of blood were used to prepare plasma for analysis of the IL-1β, TNF-α, IL-6, IL-17A, IL-12p70, IL-10, and IFN-γ levels using the Electrochemiluminescence U-PLEX Biomarker Group 1 (Mouse) Multiplex Assays (MSD, Rockville, MD, USA). Two hundred microliters of blood were used for real-time polymerase chain reaction (RT-PCR).

Part of liver, lung and spleen tissues were collected and fixed in 10% neutral buffered formalin. The remaining liver lung and brain were weighed and homogenized for bacterial load evaluation.

### 3.4. Histopathology Examinations

For histopathology examinations, samples were fixed in 10% neutral buffered formalin, embedded in paraffin, cut in 2–4μm-thick slices, and stained by hematoxylin and eosin (H&E).

### 3.5. Reverse Transcription and Real-Time Polymerase Chain Reaction (RT-PCR)

Total RNA was extracted from the blood with TRIzol reagent (Aidlab Biotech, Beijing, China). The total RNA (500 ng) in each sample were subjected to cDNA synthesis using M-MLV Reverse Transcriptase (Promega, Madison, WI, USA). RT-PCR was performed with QuantStudio 6 Flex (ABI, Foster City, CA, USA) using BioEasy SYBRGreen master mix (Bioer Technology, Hangzhou, China) according to the manufacturer’s recommendations. The transcriptional levels of the target mRNA were normalized to β-actin. Primers for the quantitation RT-PCR were as follows: CRAMP-F, GCTGTGGCGGTCACTATCAC; CRAMP-R, TGTCTAGGGACTGCTGGTTGA; S100A8-F, GGAAATCACCATGCCCTCTA; S100A8-R, TGGCTGTCTTTGTGAGATGC; lipocalin-2-F, CACGGACTACAACCAGTTCG; lipocalin-2-R, TGATGTTGTCGTCCTTGAGG; β-actin-F, CACTGCCGCATCCTCTTCCTCCC; β-actin-R, CAATAGTGATGACCTGGCCGT.

### 3.6. Flow Cytometric Analysis

Flow cytometric analysis was performed as described, with some modifications [26]. Samples of peripheral blood (0.2 mL) from il17a+/+ and il17a−/− mice were collected from a tail vein in the presence of EDTA as an anticoagulant. The red blood cells (RBS) were removed by incubation with lysing buffer (BioLegend, San Diego, CA, USA) at room temperature for 5 min. The white blood cells (WBC) were collected by centrifugation at 300× *g* at room temperature for 10 min and washed once with PBS. The Fc receptors were blocked with anti-murine CD16/CD32 at 4 °C for 30 min to prevent nonspecific binding. The cells were then stained with phycoerythrin (PE)-conjugated anti-mouse Ly6G (BioLegend, San Diego, CA, USA) and fluorescein isothiocyanate (FITC)-conjugated anti-mouse F4/80 (BioLegend, San Diego, CA, USA), and subsequently incubated at 4 °C for 60 min in the dark. After staining, the cells were washed with 2 mL of flow cytometric buffer and centrifuged at 300× *g* at 4 °C for 10 min. The cells were resuspended in 500 μL of flow cytometric buffer, after removing the cell supernatant. All samples were acquired on a cytoflex flow cytometer (Backman, Indianapolis, IN, USA), and data were analyzed using CytExpert software version 2.2 (Backman, Indianapolis, IN, USA).

### 3.7. Statistical Analysis

All assays were repeated at least three times. All data are expressed as the mean ± standard deviations. Unless otherwise specified, the data were analyzed using two-tailed, unpaired *t*-tests. The survival rates were analyzed with a log-rank test, using GraphPad Prism 6 (GraphPad Software, La Jolla, CA, USA). For all tests, *p* < 0.05 was considered statistically significant.

## 4. Conclusions

The present study demonstrated that IL-17A does not contribute to the severe inflammation, although it may play a minor role for bacterial clearance through the induction of antimicrobial proteins and the promotion of neutrophil recruitment. The results also indicate that the other signaling but not IL-17A induced by NLRP3 induction was responsible for the severe inflammation during STSLS.

## Data Availability

The data presented in this study are available in article.
